# Association of Intestinal Microbial Dysbiosis With Chronic Obstructive Pulmonary Disease

**DOI:** 10.7759/cureus.19343

**Published:** 2021-11-07

**Authors:** Fariha N Ananya, Md Ripon Ahammed, Michael M Fahem, Sunam Kafle, Mahima Viswanathan, Darshi Desai, Radhika Akku, Faryal Khan, Tabata E Hernandez, Supreet K Bala, Shivam Gulati, Natalia Martin, George D Yatzkan, Javier Pérez-Fernández

**Affiliations:** 1 Respiratory Medicine, Dhaka Medical College and Hospital, Dhaka, BGD; 2 Research & Academic Affairs, Larkin Community Hospital, South Miami, USA; 3 Cardiology, National Institute of Cardiovascular Diseases, Dhaka, BGD; 4 Internal Medicine, Larkin Community Hospital, South Miami, USA; 5 Pulmonary Medicine, Larkin University School of Medicine, Miami, USA; 6 Intensive Care Solutions, Larkin University School of Medicine, Miami, USA

**Keywords:** association of copd with pud, mucosal immunity, pathophysiology of copd, gut-lung axis, gut dysbiosis, copd

## Abstract

Normal gut flora plays various beneficial roles for the human body, including the protection against inflammatory states and mucosal viral infections. It also influences the immune system of the body. The metabolites produced by the gut bacteria control local and other systemic organs' immune functions like the lungs and brain, playing a role in their response to acute and chronic illnesses. Probiotics have shown beneficial effects on lung health. On the contrary, dysbiosis is associated with several diseases, including asthma, chronic bronchitis, emphysema, allergies, and other acute viral infections. By altering the diet of patients with respiratory diseases like patients with chronic obstructive pulmonary diseases (COPD), we may be able to mitigate their conditions. This literature review aims to discuss the mechanisms altering the gastrointestinal flora, the pathophysiology of gut and lung axis, the role of diet in gut microbe health, and the association of COPD with gut dysbiosis and peptic ulcer disease (PUD). We have extracted the data from PubMed and Google Scholar, consisting of review articles, case-control studies, and animal studies. The studies showed an association between gut microbes and different lung diseases. It is found that gut dysbiosis not only disrupts intestinal immunity but may also facilitate the development of COPD. Present studies also show an increased seroprevalence of *Helicobacter pylori* in patients with COPD. The strategies that can improve lung functions, especially in COPD patients, include prebiotics and probiotic supplementation to a diet more balanced than the current average American diet.

## Introduction and background

Chronic obstructive pulmonary disease (COPD) is an inflammatory condition of the respiratory system requiring regular visits to the clinic and hospital due to acute exacerbations [1]. Because of its high prevalence, morbidity, and mortality, it has been a major health problem worldwide [2]. The global burden of COPD is estimated to be 4-5% and is predicted to be the third leading cause of death worldwide [3]. Chronic airway inflammation and constriction of the bronchial tree limit the amount of air entering the alveoli, present in the pathophysiology, and exacerbation of several diseases such as chronic bronchitis, emphysema, asthma, and even cystic fibrosis [1,4]. In recent studies, it has been implicated that diet and nutrients that affect the gut microbiota and their metabolites (metabolome) influence the mucosal immune system of the lung [5]. It is hypothesized that the ‘gut-lung axis’ plays a role in regulating inflammation in acute and chronic respiratory diseases, such as COPD [5].

To regulate the tissue and immune homeostasis the microbiota is in a mutual association with the host executing significant favorable activities for the body, and one of them is the fermentation of dietary components which yields some metabolites [6]. One of the roles of these metabolites is to signal molecules for achieving immune and tissue homeostasis. Among the metabolites, short-chain fatty acids (SCFAs) have a small part in regulating the immunomodulatory function, and the role of other metabolites must be assessed [7]. Gut microbiota also defends against pathogens by signaling the immune system's innate and adaptive pathways. Any alteration in the distribution of the gut microbiota due to changes in diet, use of antibiotics, or genetics leads to decreased immune response [6]. This gut dysbiosis is associated with abnormal inflammatory conditions in the airways, including asthma and COPD [6]. Reduced range of gut microbes has been seen in cigarette smokers and is also considered as one of the factors involved in the pathogenesis of COPD [3]. Further, studies have shown that a diet without fiber leads to malnourishment of the microbiota, causing gut dysbiosis and later chronic inflammation both local and systemic [3]. Thus, maintaining the gut microbiota and its metabolites can be an important mechanism to manage patients with lung diseases [6-8]. Further study of this cross-talk between the gastrointestinal (GI) and pulmonary defense mechanism is essential to understand its effect on lung health, and plan future treatment strategies for managing lung diseases [4]. The objective of our study is to discuss the pathophysiology of the gut-lung axis, the mechanism altering the gut flora, and the consequences of GI tract dysbiosis on COPD.

## Review

Methodology

We searched PubMed and Google Scholar to screen for studies that established an association between airway diseases and intestinal microbiota. Our inclusion criteria were studies published in the English language, focusing only on gut microbiota, and outcomes of gut microbial dysbiosis on lung disease, primarily COPD. Both human and animal studies were included. Our exclusion criteria were studies focusing only on lung microbial dysbiosis. We extracted such articles. Data were systematically extracted using Microsoft Excel spreadsheet. The studies were reviewed in detail and subsequently analyzed by all the team members. 

Results

We found seven original studies that were relevant to our review question. Out of these one was prospective cohort study, one was case-control, two were observational studies, and three were animal studies. The summary of the studies included in our review is shown in Table 1. 

**Table 1:** Summary of the characteristics and outcomes of our included studies AECOPD, acute exacerbation of chronic obstructive pulmonary disease; GI, gastrointestinal; HIV, human immunodeficiency virus

Author	Year of publication	Type of study	Outcome/conclusion
Verhulst et al. [9]	2008	Prospective cohort study	This study demonstrated that concentration of anaerobic bacteria and antibiotic use have a significant association between wheezing in infants, excluding the *Clostridium* bacterium which had shown to be protective of wheezing
Siva et al. [10]	2013	Case-control study	The incidence of peptic ulcer disease, with or without dysbiosis due to *Helicobacter pylori*, is independently related to the detriment in measured spirometry indices in patients with varying severity of COPD
Sun et al. [1]	2019	Observational study	The study of sputum and feces of 15 subjects with AECOPD showed dynamic relation between gut-lung microbiota
Mahooti et al. [11]	2019	Animal study	The mice that received *Bifidobacterium bifidum* had developed a strong innate and adaptive immune response, which suggests supplementation of this bacterium might help to strengthen multiple immune strategies to combat mucosally transmitted microbes such as influenza virus
Yang et al. [12]	2020	Observational study	In HIV, the oral microbiota could be used as a biomarker for lung function, and its disturbance could contribute to COPD pathogenesis
Jang et al. [5]	2021	Animal study	A high-fiber diet decreases the pathological changes associated with emphysema progression and the inflammatory response in the mice exposed to smoking
Lai et al. [13]	2021	Animal study	Gut microbiota composition significantly affects cigarette smoking-induced COPD development, and fecal microbiota transplantation ameliorates COPD pathogenesis by inhibiting lung inflammation

The cohort study by Verhulst et al. suggested that clostridium might have a protective role in wheezing among the infant population [9]. In contrast, the increased concentration of other anaerobes and antibiotic use has a significant association with an increase in wheezing [9]. A case-control study by Siva et al. showed that in COPD patients with peptic ulcer disease (PUD) and gut dysbiosis in the form of *Helicobacter pylori*, there was a significant, independent relationship between the presence of PUD and the deterioration of measured spirometry indices [10]. One longitudinal study done in human immunodeficiency virus (HIV) patients affected with lung disease showed that the microbiota of the mouth could be used as a biomarker of COPD pathogenesis in this population [12]. These conclusions were corroborated in a study by Sun et al. who examined the sputum and feces of patients with acute exacerbation of COPD and found a dynamic relation between the microbiota of the lung and gut axis [1]. Animal studies demonstrated that gut microbiota and a high-fiber diet have some role in the pathogenesis of COPD in the lungs of mice exposed to smoking [5,13]. These findings extrapolate upon the relationship demonstrated by earlier studies and provide interesting insight into future therapeutic modalities involving gut health in respiratory disease.

The gut-lung axis

The gut-lung axis, referring to the interrelation of the two organ systems in immune homeostasis and susceptibility to disease, has been shown to be affected by the health and function of the intestinal microbiota.

Studies have described our primary individual intestinal microbiota as reflecting the maternal hand-over of 'seed ecology' species at birth [14,15], with the landscape being further influenced by subsequent dynamic interactions with the surrounding environment such as diet, lifestyle, disease, and antibiotic use. This developmental trajectory of the microbiome modulates our individual metabolic phenotype and greatly influences one’s biochemistry and disease susceptibility [16].

Further literature expands, as described earlier, upon the mutual association of the microbiota with the host in immune homeostasis, such as fermentation of dietary components for producing nutrients, vitamins, and metabolites, in return for which they benefit from the balanced nutrient-rich microenvironment of the host. Per Chunxi L et al., microbes protect against various pathogens by signaling the immune system's innate and adaptive arms. Any change in the composition leads to decreased immune response [17].

A large number of factors have been found to come into play in the intestinal immune system - invading pathogens must be met with an efficient response by immune cells, while also sparing the normal commensal flora. In a study by Josefowicz et al., CNS1/ mice developed a Th2-type-driven inflammation in the GI tract, indicating that Foxp3(+) regulatory T-cells (Tregs) are involved in this attempt at maintaining homeostasis [18].

Local Treg cell differentiation and function were also found to be stimulated by the gut flora per studies by Atarashi et al. [19] and Geuking et al. [20] who observed that germ-free mice have a lower number of Treg cells in the intestinal lamina propria. In this environment, the researchers found a bacterial-antigen-independent manner of pTreg induction in the small intestine, and they postulated that there was a bacterial-antigen-independent mode of pTreg induction in the small intestine. A theory of division of labor between the lamina propria of the large and small intestines emerged and was supported by the findings of Sangwon V Kim et al. [21], which indicated that the G protein-coupled receptor GPR15 was key in Treg cell enlistment to the large intestine, but not to the lamina propria of the small intestine. Per Atarashi et al. some of the specific bacteria responsible for this induction of pTreg cells in the lamina propria of the colon have been identified as *Clostridium* spp, especially cluster IV and XIVa [19].

There are more mechanisms including mucus layer, epithelial antimicrobial proteins, and IgA released by lamina propria plasma cells to reduce bacterial-epithelial invasion. In addition, the intestinal microbiota is also shown to be a key factor in the modulation of the host’s ability to manage and control inflammation. Induced B and T cell subsets circulate back to mucosal locations via the lymphatic and circulation, where B cells develop into IgA-secreting plasma cells. As a result, the gut microbiota influences the host's mucosal and systemic immunity [22].

**Figure 1:**
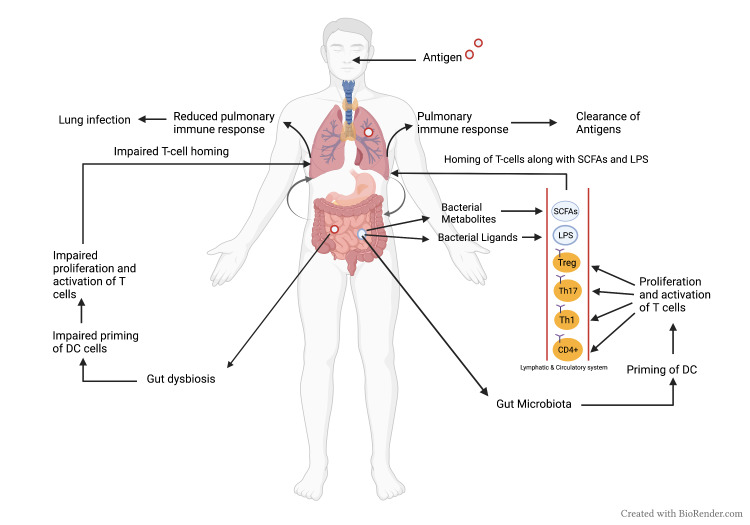
The gut-lung axis A model showing the gut-lung axis and the gut microbiota's regulatory impact on lung function immune reaction by priming DCs and then proliferation and activation of T-cells in response to antigens. Gut dysbiosis results in impaired T-cell proliferation and activation, thus causing a reduction in lung immune reaction DCs, dendritic cells; SCFA, short-chain fatty acids; LPS, lipopolysaccharides

Intestinal dysbiosis causes dysregulation of lung immune response by increasing inflammatory markers and T cells dysregulation ultimately increasing mortality from respiratory infections [23]. There is a hypothesis according to which all mucous tissues are interconnected and the activation of immune cells of gut mucosa can ultimately cause the distant immune cells such as lungs to be activated as well [24]. Similarly, the metabolites of the microbiota that are absorbed from gut mucosal tissue such as SCFAs bind to the receptors of the lungs and activate the immune cells [23,24] (Figure 1).

Altered mucosal immunity in the setting of gut dysbiosis

The gut microbiome of the average adult comprises the Firmicutes (such as *Lactobacillus*, *Clostridium*, and *Bacillus*), the Bacteroidetes (such as *Bacteroides*), and a lower concentration of the Actinobacteria (such as *Bifidobacterium*), and Proteobacteria (such as *Escherichia*). Each has been found to participate in various functions of the immune response, detoxification, digestion, metabolism, energy production, and the synthesis of useful compounds [16]. This microbiota-host interplay is tightly regulated and has been shown to be directly involved in developing and priming the immune system and also in controlling the infection [25]. Host immune system regulation and cross-talk by commensal bacteria and their components have been found to occur in animal models by mechanisms such as the production of lysozyme by epithelial barrier Paneth cells, which decreases pathogenic bacteria colonization by NOD2 detecting [26], and fermentation of food fibers to create SCFAs, which stimulate immune cell secretion of anti-inflammatory cytokines IL-10 and IL-21 [27,28]. Cell wall components such as Murein lipoprotein also induce immunoglobulin G (IgG) production [29], and antigens/pathogens are removed by primed macrophages and dendritic cells. Further, selective polarization into proinflammatory M1 phenotype by *Bacteroides fragilis* [30] has been found to enhance pathogen clearance.

In dysbiosis, overgrowth of dominant, usually pathogenic bacteria such as *Clostridium difficile* disrupts the normal action of gut flora on infection control as described. With respect to lung diseases, further study is required into whether observed microbiome alterations are causal causes or the effect of disease and if/how changes contribute to disease progression [29].

Immune homeostasis in the host is also maintained by the microbial components. Researchers have postulated a 'shared mucosal response,' in which the GI mucosa regulates immune responses at distal mucosal sites (e.g., the lungs), or vice versa, by the migration of preprogrammed lymphoid cells and/or inflammatory mediators. This has been demonstrated by Czerkinsky C et al. [31]. Another study by Noverr MC et al. found that disrupting the normal gut microbiota in inbred mice can cause local fungal overgrowth, which can lead to experimental asthma when the mice are exposed to fungal spores later in life. Increased levels of eosinophils, IgE, IL-5, and IL-13, as well as goblet cell metaplasia, characterize this allergic airway illness [32]. Such studies seem to demonstrate a well-established two-way connection with demonstrated mechanisms between the gut flora and distal organs with respect to immunity. Further research into these mechanisms may help shape holistic therapies in respiratory disease involving gut health and targeted respiratory interventions as well.

COPD and gut dysbiosis

**Figure 2:**
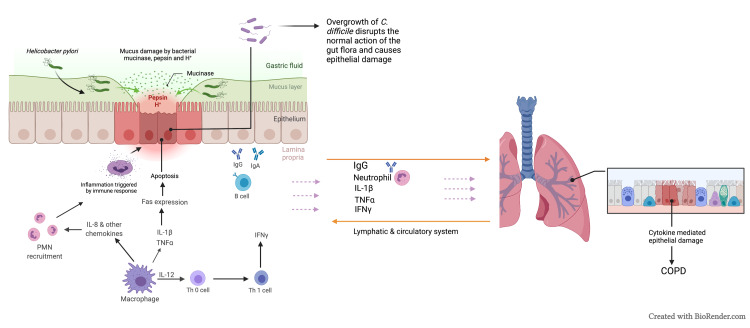
Representation of the interaction between the lungs and the intestine in disease settings. Microbial dysbiosis within the intestine results in exaggerated immune reactions to the microbiome, as well which may lead to loss of integrity and function of epithelial barrier in both the intestines and the lungs COPD, chronic obstructive pulmonary disease; IL-1, interleukin 1; TNF, tumor necrosis factor; IgG, immunoglobulin G; IFN, interferon

Nutritional changes, mucin consumption, antimicrobial generation, anaerobic/aerobic respiration, and metal utilization are all thought to have a role in resistance to colonization by enteric pathogens [25]. A key conclusion drawn from these findings is that a decrease in total bacterial diversity may negatively affect host health. This gut dysbiosis, as discussed previously and indicated in several studies, is associated with inflammatory conditions in the airways, including asthma and COPD [6]. Clinical research has demonstrated that cigarette smoking, considered as one of the factors involved in the pathogenesis of COPD, also decreases the range of the gut microbes, and this development, in turn, is also involved in the pathogenesis of COPD [33] (Figure 2).

COPD in Helicobacter pylori infections

A common cause of gut dysbiosis is *H. pylori*, causing PUD. A study performed to assess the seropositivity of *H. pylori* on 80 subjects classified into two groups, COPD and non-COPD patients, demonstrated statistically significant results showing that seropositivity was higher in COPD patients than non-COPD patients [34]. Sze MA et al. employed an immunoassay to determine *H. pylori* IgG antibody titers in serum samples from 4765 individuals with mild-to-moderate COPD. Researchers discovered that *H. pylori* infection was linked to lower lung function, later systemic inflammation, and an increased risk of cardiovascular death in patients with COPD, suggesting that the bacterium had a negative influence on lung growth [4]. There is a strong indication here that the development of gut dysbiosis is related to the development of gut disease as well as exacerbation of a chronic airway disease such as COPD, with a key factor being inflammation. Looking further into whether the reverse also holds true would doubly illuminate this lung-gut connection and could be extrapolated to devise therapeutic benefit.

Role of oropharyngeal dysbiosis in lung disease

While the role of gut microbiome alteration on lung disease has been widely studied, limited studies are available that explore the role of oral microbial dysbiosis on lung injury. A recent study conducted on HIV-positive patients described the impact of oropharyngeal dysbiosis on pulmonary disease [12]. HIV-positive status causes a compromise in the immune system of a patient that ultimately affects the oral microbial flora. Patients with HIV were found to have increased proportions of unusual bacteria such as *Veillonella*, *Streptococcus*, and *Lactobacillus* in their oropharynx. These alterations of oral microbiota were linked with systemic inflammation and the incidence of COPD in the population [12]. While the role of oropharyngeal dysbiosis in immunocompromised patients is studied, there are limited data available relating to oropharyngeal dysbiosis and lung injury in healthy populations.

Role of diet in gut microbe health

The authors have reviewed many studies on inflammation of the gut and systemic disease which have determined that a decrease in total bacterial variety could be harmful to the host's health [35-37], and which signify that distinct gut microbiota members play roles in diverse aspects of health. During our review, we found that one of the hallmark features of maintaining such a diverse healthy gut microbiome appears to be one’s diet, as we now discuss.

Fiber as a prebiotic supplement

The prebiotic effect of dietary fiber has been shown to determine gut flora composition, whose modulation of immune response and metabolic functions has multisystem effects [5]. SCFAs such as acetate, propionate, and butyrate are among the metabolites derived by dietary fiber fermentation. Their ratio in the gut at 6:3:1 is found to relate to the composition of the microbiome, carbohydrate composition in the diet, and intestinal transit time in a study by Macfarlane S and Macfarlane GT [38]. SCFAs help preserve colonic epithelial integrity, regulate host energy balance, decrease colonic inflammation, and induce apoptosis in colon cancer cells by serving as the principal energy source for colonocytes [27,28].

Apart from the local environment of the colon, the immune response of other organs can be influenced by the micro-aspiration of gut bacteria or the passage of sensitized immune cells through the lymph or circulation, further downstream, suggesting a multisystem effect [39]. This effect is indirectly influenced by a high-fiber diet. Anand S et al. and Jang YO et al. independently noted that a diet without fiber leads to malnourishment of the microbiota, causing the effects of gut dysbiosis at the mucosal level, later leading to local and systemic chronic inflammation [5,39].

In a recent randomized prospective study by Hannah C Wastyk et al., the effects of plant-based fiber versus fermented food supplementation to the diets of healthy adults were observed. Despite steady microbial community diversity, a high-fiber diet boosted microbiome-encoded glycan-degrading carbohydrate active enzymes (CAZymes) within 10 weeks, and three unique immunological trajectories in high-fiber consumers matched baseline microbiota diversity [40]. These results suggest that optimal benefits from a high-fiber diet may be achieved with consistency over a longer period of time. Further, direct supplementation of commensal bacteria may optimize carbohydrate digestion in a high-fiber diet and could be a focus of future research.

Fermented plant food as a probiotic supplement

Natural substances such as probiotics and phytochemicals found in fermented plant products have been shown to have anti-infective, antioxidative, anti-inflammatory, anti-angiogenic, and anticarcinogenic biologic activities [41]. Fermentation has been shown to enhance the amount of peptides, amino acids, vitamins, minerals, and antioxidants in foods [42-45]. The demonstrated effects of fermented soy, cabbage, and berries were reviewed as follows.

Fermented soy products have exhibited anti-inflammatory activity by reducing free radical production, suppressing nuclear factor kappa B signaling, and inhibiting cyclooxygenase-2 and inducible nitric oxide synthase expression in a study conducted on rats [46]. Other studies on fermented cabbage and berries have also demonstrated significant anti-inflammatory, immune, and gut-microbe-modulating effects [47-50].

Per Hannah C Wastyk et al. studying the effects of plant-based fiber versus fermented food supplementation, the fermented foods were found to achieve the primary endpoint of decreased inflammatory markers as well as a steady increase in gut microbe diversity, unexpectedly ahead of the study arm on fiber supplementation [40]. 

These findings suggest that probiotic supplementation could have a role in combating inflammation in the gut, which could then prevent or mitigate exacerbations in lung conditions such as COPD.

Diet and nutrition in COPD

Inadequate nutrition is associated with many chronic inflammatory diseases, including respiratory [51-55] via p38 mitogen-activated protein kinase signaling, among other methods. Nutrition by definition stems from the diet, a modifiable lifestyle factor characterized by the types of food routinely consumed by an individual. Studies have proven that the Western diet, typically characterized by processed meat/sugar, refined grain, trans-fat, and alcohol, is often nutrient deficient [56,57]. Regular incorporation of these foods in the diet has been associated with worsening systemic inflammation, chronic cough with expectoration, and progressive airflow obstruction characteristic of COPD in susceptible individuals. It has also been directly associated with increased COPD risk in men and women [33,58]. In comparison, an inverse relationship has been found to exist between diets rich in fruit/vegetables, whole grains, whole-fat dairy, and including adequate hydration, and the risk of COPD [33,58]. According to a study by S O Shaheen et al., these are components of what is known as a ‘prudent diet’ [59] with health benefits. Moving forward, research-backed recommendations and widespread public health education programs could help encourage the incorporation of elements of the ‘prudent’ diet and fermented food items into the average American diet.

Limitations and future implications

The exact mechanism of COPD due to gut dysbiosis is not clear, and there are many theories approving the effect of gut dysbiosis on COPD. Factors causing/exacerbating COPD in gut dysbiosis may have similar effects on other respiratory diseases as well. Many of the articles we reviewed in this study were not clinical trials based on human beings. Future implications of the topic in randomized controlled trials will be helpful and more evident. Also, modification of policies and investment at the level of local, state, and federal government would be required to improve availability and access to the ‘prudent diet’ and fermented foods. However, in the long term, this may very well show a reduction in overall respiratory, GI, and cardiovascular disease burden, currently a regular drain on medical resources. This discussion is beyond the scope of this article at present but provides a promising prospect for further work in the fields of medicine and public health.

## Conclusions

Chronic intestinal dysbiosis may lead to exacerbation of preexisting COPD or may facilitate the development of COPD in a healthy lung. The microbiota of the intestine plays a significant role in lung immune reactions by producing metabolites like SCFAs, and priming immune cells that travel through the lymphatic and circulatory system, acting on the lung mucosa. Thus, disruption of the microbiota in the intestine termed dysbiosis not only affects the intestinal immunity but may also facilitate disease development in the lung. The high plant-based fiber content in the diet and fermented foods have been proven to be beneficial for gut microbiota-metabolite modulation. This may also be beneficial for the prevention and treatment of COPD.
